# Editorial: Reviews in radiation oncology

**DOI:** 10.3389/fonc.2023.1283431

**Published:** 2023-09-06

**Authors:** David Y. Lee, Ganapasam Sudhandiran, Sunil D. Sharma

**Affiliations:** ^1^ Department of Internal Medicine, Division of Hematology/Oncology, Section of Radiation Oncology, University of New Mexico School of Medicine and Comprehensive Cancer Center, Albuquerque, NM, United States; ^2^ Department of Biochemistry, University of Madras, Chennai, India; ^3^ Radiological Physics and Advisory Division, Bhabha Atomic Research Centre, Mumbai, India

**Keywords:** PSMA, PET/MRI, radiotherapy, oligometastasis, meningioma, cardiac implantable electronic device (CIED), nasopharynx, HDAC inhibitor

## Introduction

This Frontiers Research Topic is focused on timely updates on the prostate and pancreas staging using novel imaging modalities, new meningioma classification, efficacy of local ablative radiation therapy in oligometastatic setting, and the effects of radiation treatment interruptions and potential strategies to overcome them.

Zhang et al. provide updated reviews on the role of prostate-specific membrane antigen (PSMA) imaging in prostate cancer staging. The authors provide data on the sensitivity and specificity of several radioactive tracers such as 18F and 68Ga – PSMA scans. As we incorporate PSMA imaging in the initial staging as well as at the time of recurrence, we have observed more opportunities for radiation therapy. PSMA scans have been particularly useful at the time of recurrence after radical prostatectomy or definitive radiation therapy. One often finds limited number of lymph nodes or skeletal metastasis which are amenable to stereotactic ablative radiotherapy (SABR) treatments (1). The advent of PSMA imaging has led to dramatic increase in the utilization of SABR treatments in prostate cancer with potential to reduce the need for androgen deprivation therapy (ADT). In addition, PSMA scans have allowed us to detect limited number of pelvic or skeletal metastasis (oligometastatic state) at the time of diagnosis with more confidence, and we have been able to deliver aggressive radiation therapy to both the primary site and the oligometastatic sites based on the results of STAMPEDE clinical trial which showed survival benefit (2).

Li et al. provide the role of PET/MRI in pancreatic cancer. PET/MRI provides greater diagnostic accuracy and staging similar to PSMA imaging for prostate cancer. From a radiation oncology perspective, the PET/MRI has the potential to more effectively incorporate SABR treatment in early stage, unresectable/borderline resectable pancreatic cancer. Often, the problem arises in target delineation with independent CT, PET, and MRI scans which are fused together in the radiation treatment planning software. Use of PET/MRI would reduce mis-registration issues and help us to precisely delineate target lesions with greater confidence. In addition, FDG-avid lesions potentially could be dose-escalated simultaneously, allowing greater dose delivery while minimizing dose to the surrounding critical structures such as the duodenum. PET/MRI imaging is also being evaluated in other cancers such as prostate and head and neck for target delineation, initial staging, and treatment response assessment (3–5). We await to see whether PET/MRI could be incorporated into routine radiation oncology practice.

Yarabarla et al. provide an update on intracranial meningioma. A new molecular classification has been incorporated for Grade 3 meningioma with TERT promoter mutation or homozygous deletion of CDKN2A and/or CDKN2B. There is also an excellent discussion on conventionally fractionated radiation therapy vs stereotactic radiosurgery (SRS) on efficacy, selection criteria and side effects.

While novel imaging modalities (PSMA and PET/MRI) have increased the use of SABR, we have limited data from clinical trials on whether such treatment leads to survival benefit in oligometastatic setting. Zhang et al. provide a wonderful update on the most recent and important clinical trials supporting the use of local ablative therapy in non-small cell lung cancer (NSCLC). The authors provide excellent discussion on the results of the three phase II trials supporting the use of SABR in oligometastatic setting (6–8). We eagerly await the results of large phase III randomized trials such as NRG-LU002. How to incorporate immunotherapy with SABR is also an important question in the field, and there are active investigations in this area. Mirzaei et al. also provide an update and guidelines on the cardiac implantable electronic device (CIED) for patients receiving proton therapy. This is relevant since many lung cancer patients with CIED receiving proton radiation therapy will need guidelines on CIED management.

Finally, Zhao et al. provide an update on the detrimental effect of radiotherapy treatment interruption on nasopharyngeal cancer. The authors provide the impact of missing 3 to 5 days of radiation on progression free survival and overall survival. Strategies to overcome potential inferior outcome include dose intensification, twice a day treatment or the addition of systemic therapy. We also note that the time to initiation of radiation therapy after surgery should be less than 6 weeks (9, 10). Ling et al. discuss the role of HDAC inhibitors for overcoming radiation therapy resistance. Several mechanisms of HDAC inhibitors are provided including downregulation of PARP expression. The combination of HDAC inhibitor and radiation therapy should be further explored in solid tumors and in the setting of radiation treatment interruption. In summary, our Research Topic has provided additional insight on the role of new imaging modalities (PSMA and PET/MRI), novel molecular classification of meningioma, role of local ablative radiation therapy in oligometastatic setting, and potential strategies to address radiation therapy interruptions.

## Author contributions

DL: Writing – original draft, Writing – review & editing. GS: Writing – review & editing. SS: Writing – review & editing.
